# Peripheral artery disease mediating the effect of metabolic syndrome related diseases on lower limb ulcers: Mendelian randomization analysis

**DOI:** 10.3389/fendo.2024.1345605

**Published:** 2024-02-15

**Authors:** Huan Wang, Zhe Zhang, Linxuan Zou, Juewei Zhang, Zhuqiang Jia, Lin Zhao, Xin Han, Xiaohong Sun, Zhen Zhang, Junwei Zong, Shouyu Wang

**Affiliations:** ^1^ Department of Orthopaedic Surgery, The First Affiliated Hospital of Dalian Medical University, Dalian, China; ^2^ Health Inspection and Quarantine, College of Medical Laboratory, Dalian Medical University, Dalian, China; ^3^ Department of Surgery, The First Affiliated Hospital of Dalian Medical University, Dalian, China; ^4^ Department of Surgery, Naqu People’s Hospital, Tibet, China; ^5^ Department of Quality Management, Dalian Municipal Central Hospital, Dalian, China; ^6^ Department of Orthopaedic Surgery, The Second Affiliated Hospital of Dalian Medical University, Dalian, China; ^7^ Department of Nursing, The First Affiliated Hospital of Dalian Medical University, Dalian, China

**Keywords:** hypertension, body mass index, hyperuricemia, type 2 diabetes, peripheral artery disease, ulcers of lower limb, Mendelian randomization

## Abstract

**Background:**

Previous observational studies have demonstrated a correlation between metabolic syndrome related diseases and an elevated susceptibility to ulcers of lower limb. It has been suggested that this causal relationship may be influenced by the presence of peripheral artery disease (PAD). Nevertheless, the precise contribution of these factors as determinants of ulcers of lower limb remains largely unexplored.

**Method:**

This research incorporated information on hypertension, BMI, hyperuricemia, type 2 diabetes, PAD, and ulcers of lower limb sourced from the GWAS database. Univariate Mendelian randomization (SVMR) and multivariate Mendelian randomization (MVMR) methods were employed to assess the association between metabolic syndrome related diseases, including hypertension, obesity, hyperuricemia, and type 2 diabetes, as well as to investigate whether this association was influenced by PAD.

**Results:**

Univariate Mendelian randomization analysis showed that genetically predicted hypertension, BMI, and type 2 diabetes were associated with an increased risk of PAD and ulcers of lower limb, and PAD was associated with an increased risk of ulcers of lower limb, but there is no causal relationship between hyperuricemia and ulcers of lower limb. The results of multivariate Mendelian randomization showed that PAD mediated the causal relationship between hypertension, obesity and ulcers of lower limb, but the relationship between type 2 diabetes and ulcers of lower limb was not mediated by PAD.

**Conclusion:**

Hypertension, BMI and type 2 diabetes can increase the risk of ulcers of lower limb, and PAD can be used as a mediator of hypertension and obesity leading to ulcers of lower limb, These findings may inform prevention and intervention strategies directed toward metabolic syndrome and ulcers of lower limb.

## 1 Introduction

Ulcers of lower limb are a prevalent condition in the field of surgery, with chronic ulcers of lower limb posing significant challenges. These ulcers exhibit prolonged healing periods or recurrent episodes following initial recovery, thereby significantly impairing individuals’ daily functioning and occupational activities. In severe cases, these ulcers may even progress to malignancy or necessitate amputation (1–4). There exists a variety of lower limb ulcers, including but not limited to diabetic foot ulcers, vascular ulcers, pressure injuries, infectious ulcers, and radiation ulcers. However, a common characteristic among these conditions is their association with ischemic lesions. Peripheral artery disease (PAD) is a prevalent etiology of lower limb ischemia (5). It is a vascular disorder primarily caused by the progression of atherosclerosis, although it can also be associated with connective tissue disease or vasculitis. It is characterized by the partial or complete stenosis of one or more arteries (6). The development of arterial stenosis resulting from atherosclerosis in PAD can contribute to the occurrence of limb skin ischemia. Several studies have indicated that PAD may be implicated in the development of ulcers of lower limb (7–9). Numerous lower limb ulcer-causing diseases often manifest with lower limb ischemia as their initial symptom, leading to an uncertain association with PAD. Numerous conditions leading to ulcers in the lower limbs often manifest with lower limb ischemia as the initial indication. The association between these conditions and peripheral artery disease (PAD) remains unclear. The specific role of PAD in facilitating lower-extremity ulcers is not yet fully understood.

Metabolic syndrome is a condition characterized by abnormal levels of protein, fat, carbohydrates and other substances in the body. It encompasses a range of complex metabolic disorders and is a risk factor for cardiovascular and cerebrovascular diseases, as well as diabetes. It is marked by various metabolic irregularities, such as obesity, high blood sugar, hypertension, abnormal lipid levels, increased blood viscosity, elevated uric acid, fatty liver, and high insulin levels, all of which contribute to the development of heart and cerebrovascular diseases and diabetes. It is evident that hypertension, obesity, type 2 diabetes, and hyperuricemia is not the standalone conditions, but rather a component of metabolic syndrome.

Several research studies have indicated a potential association between metabolic syndrome related diseases such as hypertension, obesity, hyperuricemia, and type 2 diabetes, and the development of lower limb ulcers (9–17). The majority of diseases linked to metabolic syndrome, which result in ulcers on the lower limbs, may typically originate from lower limb ischemia. However, the precise mechanisms through which chronic diseases contribute to the occurrence of these ulcers remain unclear. What’s more, the etiology of lower limb ulcers resulting from metabolic dysfunction is attributed not only to lower limb ischemia, but also to a reduction in the secretion of stem cell factor. A study demonstrated that the delayed closure of skin wounds in individuals with metabolic syndrome is associated with a deficiency of stem cell factor (SCF) in keratinocytes (18).

PAD is a prevalent condition that leads to ischemia in the lower extremities as a result of lower extremity artery disease. However, the extent to which these diseases are influenced by PAD in relation to the incidence of ulcers of lower limb remains uncertain. It is postulated that PAD may serve as a mediator or potential mechanism through which chronic illnesses contribute to the development of lower limb ulcers. Therefore, a comprehensive comprehension of the pathological mechanisms linking chronic diseases and lower limb ulcers can greatly inform the management and prevention of these ulcers in clinical settings and public health settings. Obesity can be assessed using a variety of methods. The World Health Organization defines obesity as having a BMI equal to or exceeding 30 kg/m^2^, although this specific threshold may vary across different populations (19).

Mendelian Randomization (MR) is a robust statistical approach employed to investigate potential causal associations between environmental factors and intricate diseases (20, 21). By utilizing genetic variants as instrumental variables, MR effectively mitigates the impact of confounding factors commonly encountered in conventional studies, thereby enhancing the credibility and validity of the findings (22, 23). Genetic variants are assigned randomly during conception, which reduces the influence of environmental factors on their associations with the outcome. In recent times, researchers have utilized Mendelian randomization (MR) methods to explore mediating pathways. By employing genetic variants that represent lifetime exposure, these methods effectively address the bias caused by measurement errors that often impede observational studies.

The objective of this research was to utilize the MR framework to examine the impact of Hypertension, Higher BMI, hyperuricemia, and type 2 diabetes on the risk of lower limb ulcers. Additionally, we sought to employ MR mediation analyses to explore the extent to which these chronic diseases may mediate the effects of PAD on the development of lower limb ulcers.

## 2 Materials and methods

### 2.1 Study design

**Figure 1:**
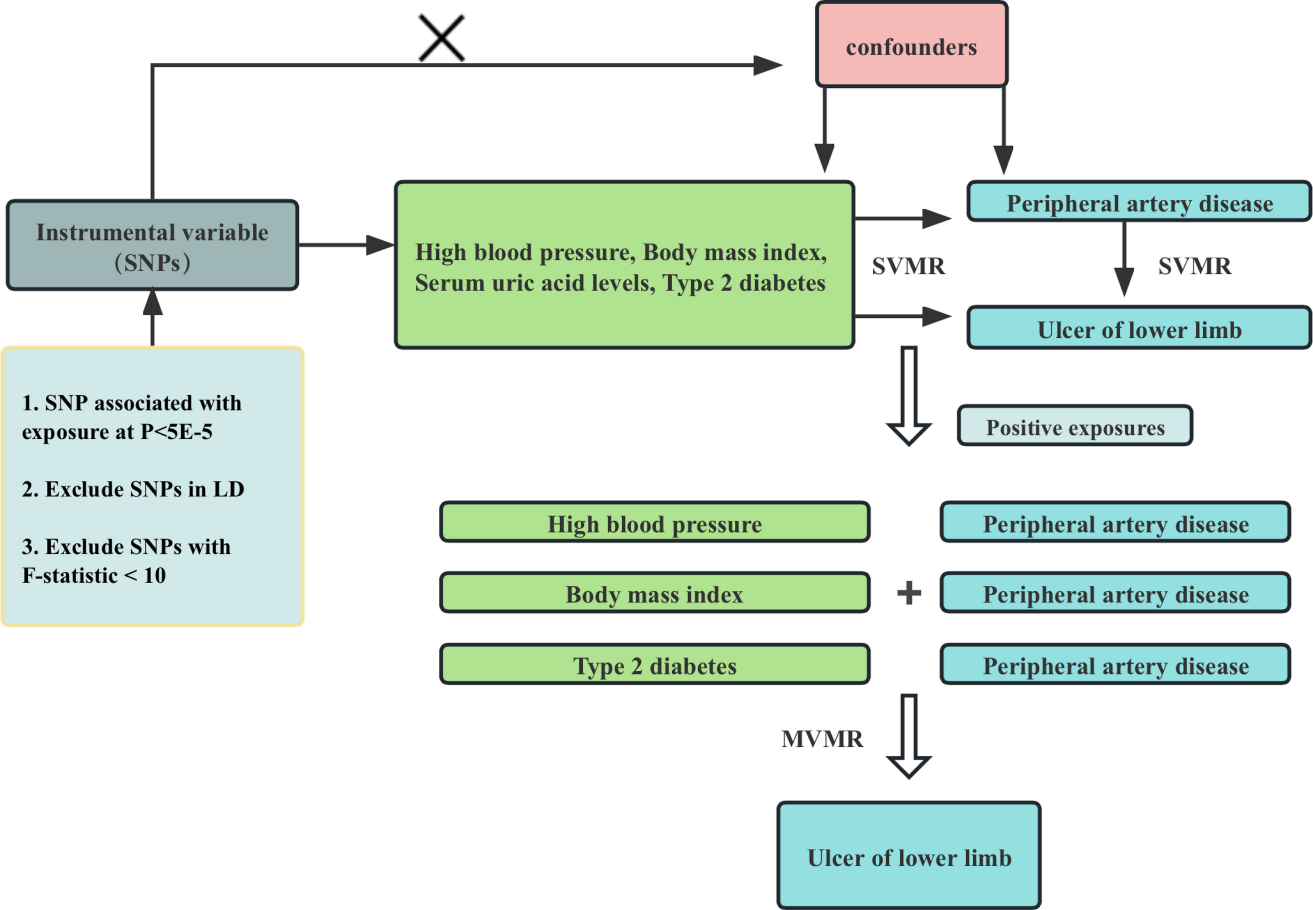
Flowchart of Mendelian randomization analysis conducted in this study. SVMR analysis investigates the effect of High blood pressure, Body mass index, Serum uric acid levels, Type 2 diabetes on Peripheral artery disease and Ulcer of lower limb, MVMR analysis evaluates the roles of Peripheral artery disease mediating the association between high blood pressure, Body mass index, Serum uric acid levels, Type 2 diabetes and Ulcer of lower limb.

All of our data comes from the genome-wide association studies (GWAS) database, a platform specifically designed to store and collate data from GWAS. These data typically include information about the genotype, phenotype, and relationships between the various diseases. These databases allow users to access and analyze data to gain a deeper understanding of the genetic mechanisms underlying disease development. The framework of the current MR Study is shown in **Figure 1**. In this study, we first used univariate Mendelian randomization to explore the relationship between hypertension, BMI, uric acid level, type 2 diabetes and ulcers of lower limb and PAD, and then used multivariate Mendelian randomization to further explore whether the causal relationship between them and ulcers of lower limb is mediated by PAD.

### 2.2 Data sources

In this MR study, summary statistics on hypertension, BMI, Serum uric acid levels and type 2 diabetes were obtained from the European Bioinformatics Institute (EBI) database, and data on PAD, and ulcers of lower limb were obtained from the Finnish database. The respective sample sizes were as **Table 1**. We acquired condensed data on the association between single nucleotide polymorphisms (SNPs) and phenotypes from GWAS conducted for each specific phenotype, all datasets included observations of men and women of European descent, and all studies that make up the GWAS have existing ethical clearances from their respective institutional review boards, including participants’ informed consent and strict quality controls; details are provided in **Table 1**.

**Table 1:** Details of the studies included in the Mendelian randomization analyses.

Phenotype	Dataset	Ethnicity	Sample size	GWAS id	PubMed ID
High blood pressure	EBI	European	407746 individuals	ebi-a-GCST90013916	34017140
Body mass index	EBI	European	359983 individuals	ebi-a-GCST90018947	34594039
Type 2 diabetes	EBI	European	38,841cases, 451,248 controls	ebi-a-GCST90018926	34594040
Serum uric acid levels	EBI	European	343836 individuals	finn-b-I9_CVD	34594039
Peripheral artery disease	FinnGen	European	7,098 cases, 206,541 controls	finn-b-I9_PAD	NA
Ulcer of lower limb	FinnGen	European	1,584 cases, 207,482 controls	finn-b-L12_ULCERLOWLIMB	NA

### 2.3 Selection of genetic instrumental variables

Genetic variants significantly associated with hypertension, BMI, type 2 diabetes, hyperuricemia and PAD (p<5×10^-5^) were selected as instrumental variables, and SNPs independence was ensured by linkage disequilibrium (LD: r^2^ = 0.001, kb = 10,000). The statistical strength (F-statistics) was calculated. An F-value greater than 10 indicated the absence of weak instrumental variable bias. The exposure and outcome data sets were finally reconciled to ensure that the effect alleles belonged to the same allele. SNPS screened through these rigorous procedures can be used as IVs for subsequent analysis.

### 2.4 Mendelian randomization design and data analysis

TwoSampleMR, MR-PRESSO and MVMR packages in R software are used for analysis. Inverse variance weighting (IVW) was used as the default method to evaluate causal estimates, and the method MR-Egger and weighted median were used for validation. The main idea of IVW method is to use Instrumental variables (IV) to study causality and deal with confounding factors. In this process, SNP is used as an instrumental variable, and its effect size (beta value) and standard error (se value) are necessary data for MR Analysis. The advantage of this approach is that if SNPS fully conform to the three principles of MR Research: association, consistency, and independence, then correct causal estimates can be derived. The MR-Egger method is a method to check the externality of instrumental variables. It evaluates the externality of instrumental variables by calculating the slope and intercept of the regression coefficient. If the slope is significantly non-zero, there is bias, that is, the instrumental variable is correlated with the error term, which requires correction using other methods. The weighted median method is a weight-based method that multiplies the effect size of each SNP by its corresponding weight, and then finds the weighted median of all SNPS as the final causal estimate. The advantage of this approach is that it can handle situations with a large number of SNPS and has less impact on outliers. However, since the choice of weights may affect the result, care needs to be taken to select the appropriate weights when using this method. A p-value less than 0.05 indicated statistical significance. To ensure the reliability of the results, sensitivity analyses (heterogeneity and pleiotropy tests), MR-Egger intercept test and leave-one-out test were performed for comparison. Finally, MR-PRESSO test was used to evaluate the presence of outlier SNPS. In univariate Mendelian randomization, the relationship between hypertension, BMI, type 2 diabetes, hyperuricemia and PAD and ulcers of lower limb, as well as the relationship between PAD and leg ulcer were explored. For MVMR, we used leg ulcer as the outcome and adjusted PAD to explore whether hypertension, BMI, type 2 diabetes, and hyperuricemia caused leg ulcer through PAD. The hypothesis validity of our MR Study is based on the following three core assumptions: (1) association hypothesis: genetic variants are strongly associated with exposure; (2) independence assumption: genetic variation is not associated with any confounders that may mediate the way from exposure to outcome; (3) Exclusion-restriction hypothesis: genetic variation may only affect the outcome through exposure. To exclude the effect of heterogeneity, the random effects model (IVW) was used as the main method, and MR-Egger and weighted median methods were used for validation.

### 2.5 Ethical approval

No ethical approval was deemed unnecessary as the current study did not involve the use of original data.

## 3 Results

The number of SNPS screened as instrumental variables significantly associated with exposure ranged from 135 to 806, with an F-statistic greater than 10 for each SNP in the study (**Table 2**).

**Table 2:** The number of SNPs and F-statistics of instrumental variables.

Phenotype	nSNPS	F-statistics
High blood pressure	567	37.77
Body mass index	806	42.73
Type 2 diabetes	502	42.51
Serum uric acid levels	583	68.95
Peripheral artery disease	136	19.49

### 3.1 Univariate Mendelian randomization was performed

#### 3.1.1 MR analysis of hypertension, BMI, type 2 diabetes, serum uric acid levels and ulcers of lower limb

**Figure 2:**
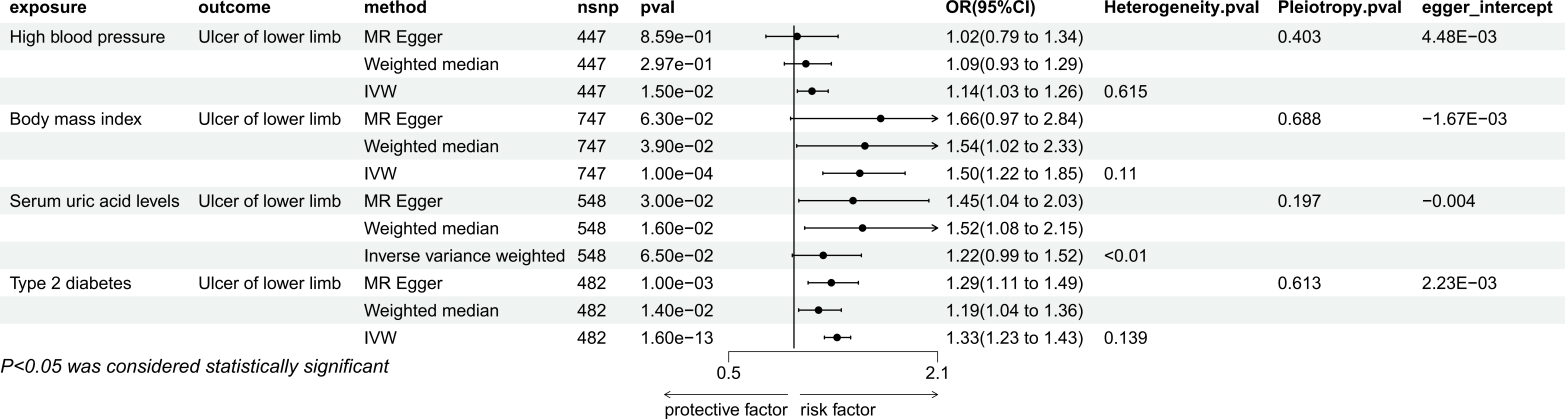
Mendelian randomization result of the effect of High blood pressure, Body mass index, Serum uric acid levels, Type 2 diabetes on Ulcer of lower limb.

IVW estimates showed that there were causal relationships between genetically predicted hypertension (P=1.50e-02, OR=1.14, 95%CI=1.03-1.26), BMI (P=1.00e-04, OR=1.50, 95%CI=1.22-1.85), type 2 diabetes (P=1.60e-13, OR=1.33, 95% CI=1.23-1.43) and ulcers of lower limb, but there was no causal relationship between uric acid levels and lower limb ulcers (P=6.50e-02, OR=1.22, 95% CI=0.00-1.52) (**Figure 2**). Among them, hypertension was not significant in MR Egger’s method and weighted median method, BMI was not significant in MR Egger’s method, but significant in weighted median method (P=3.90e-02, OR=1.54, 95%CI=1.02-2.33). Type 2 diabetes remained significant in both methods. For positive exposure, in the sensitivity analysis, there was no heterogeneity and pleiotropy, and the leave-one-out method also showed no SNPs that independently drove their causal relationship with ulcers of lower limb (**Supplementary Document 1**). The egger intercept did not deviate significantly from 0. The MR-PRESSO results showed no outlier SNPs (**Supplementary Document 2**).

#### 3.1.2 MR analysis of hypertension, BMI, type 2 diabetes, serum uric acid and PAD

**Figure 3:**
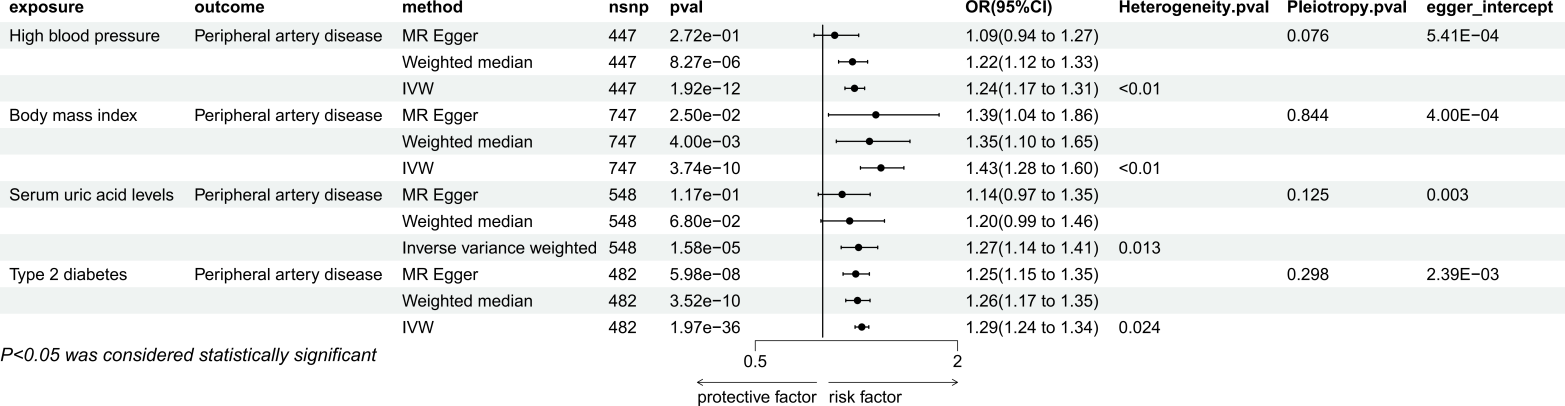
Mendelian randomization result of the effect of High blood pressure, Body mass index, Serum uric acid levels, Type 2 diabetes on Peripheral artery disease.

IVW method showed that there were causal relationships between genetically predicted hypertension (P=1.92e-12, OR=1.24, 95% CI=1.17-1.31), BMI, (P=3,74e-10, OR=1.43, 95%CI=1.28-1.60), type 2 diabetes (P=1.97e-36, OR=1.29, 95%CI=1.1.24-1.34), Serum uric acid (P=1.58e-05, OR=1.27, 95% CI=1.14-1.41) and PAD (**Figure 3**). Among them, hypertension was not significant in MR Egger method, but were significant in weighted median method (P=8.27e-06, OR=1.22, 95%CI=1.12-1.33), while BMI and type 2 diabetes were still significant in the other two methods(BMI: MR Egger, P=2.50e-02, OR=1.39, 95%CI=1.04-1.86, weighted median: P=4.00e-03, OR=1.35, 95%CI=1.10-1.65; type 2 diabetes: MR Egger, P=5.98e-08, OR=1.25, 95%CI=1.15-1.35, weighted median: P=3.52e-10, OR=1.26, 95%CI=1.17-1.35), Serum uric acid was not significant in MR Egger’s method and weighted median method. In sensitivity analysis, they are all heterogeneous, but no pleiotropy. The leave-one-out method also showed that there was no SNP that independently drove their causal relationship with PAD (**Supplementary Document 1**), and the egger intercept did not deviate significantly from 0. The results of MR-PRESSO showed the presence of outlier SNPS, and the results were still significant after excluding the outlier SNPS (**Supplementary Document 2**).

#### 3.1.3 MR analysis of PAD and lower extremity ulcer

**Figure 4:**
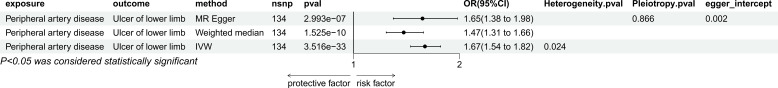
Mendelian randomization result of the effect of Peripheral artery disease on Ulcer of lower limb.

The genetic prediction of PAD to increase the risk of lower extremity ulcer (**Figure 4**) was validated in IVW (P=3.516e-33, OR=1.67, 95%CI=1.54-1.82), MR Egger (P=2.993e-07, OR=1.65, 95%CI=1.38-1.98) and weighted median methods (P=1.525e-10, OR=1.47, 95% CI=1.31-1.66). In sensitivity analysis, there was heterogeneity, but no pleiotropy. The leave-one-out method also showed that there was no SNP that independently drove their causal relationship with PAD (**Supplementary Document 1**), and the egger intercept did not deviate significantly from 0. MR-PRESSO results showed no outlier SNPS (**Supplementary Document 2**).

### 3.2 Multivariate Mendelian randomization

#### 3.2.1 Mediation study between hypertension and lower extremity ulcer

**Figure 5:**
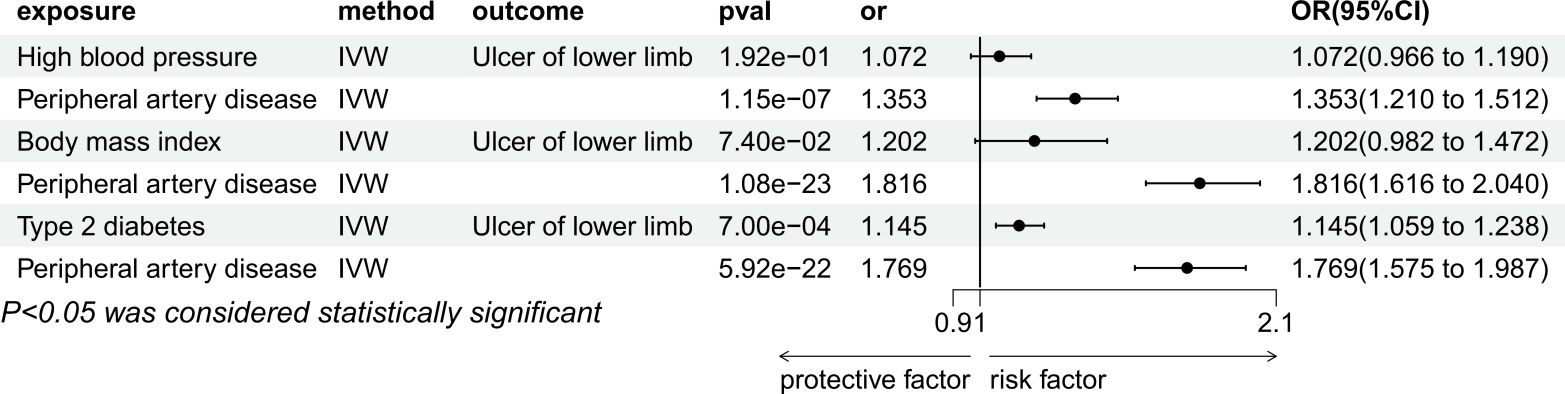
Multivariable MR result of causal relationships of High blood pressure, Body mass index, Type 2 diabetes and Peripheral artery disease on T2DM.

In a multivariable Mendelian randomization analysis, the study investigated the correlation between hypertension and ulcers of lower limb while controlling for peripheral artery disease (PAD). The findings revealed that the link between hypertension and ulcers of lower limb lost statistical significance (P=1.92e-01, OR=1.07, 95%CI=0.97-1.19). However, the association between PAD and leg ulcers remained statistically significant even after adjusting for PAD (P=1.15e-07, OR=1.35, 95%CI=1.21-1.51), as illustrated in **Figure 5**.

#### 3.2.2 Mediation study between BMI and lower extremity ulcer

In the multivariate Mendelian randomization analysis conducted, the relationship between PAD and leg ulcers remained statistically significant (P=1.08e-23, odds ratio [OR]=1.82, 95% confidence interval [CI]=1.62-2.04) after adjusting for PAD. However, the association between BMI and ulcers of lower limb became non-significant (P=7.40e-2, OR=1.20, 95%CI=0.98-1.47) in **Figure 5**.

#### 3.2.3 Mediation study of type 2 diabetes mellitus and lower extremity ulcer

Multivariate Mendelian randomization analysis of type 2 diabetes and PAD with ulcers of lower limb after adjusting for PAD showed that both type 2 diabetes (P=7.00e-04, OR=1.15, 95%CI=1.06-1.24) and PAD (P=5.92e-22, OR=1.77, 95%CI=1.57-1.99) were significantly associated with ulcers of lower limb in **Figure 5**.

## 4 Discussion

Our study utilizes extensive GWAS data to examine the impact of genetically predicted conditions including hypertension, hyperuricemia, higher body mass index, and type 2 diabetes on lower limb ulcers within the MR framework. The etiology of ulcers of lower limb encompasses a wide range of exposure factors. Many of the aforementioned exposures are prevalent chronic conditions, which exhibit a significant interrelationship. Given that chronic ischemia plays a pivotal role in the development of ulcers of lower limb, it is reasonable to posit that PAD serves as a shared mediator for these exposure factors. The findings indicate that hypertension and higher BMI contribute to lower limb ulcers through the involvement of PAD. However, the cause of ulcers of lower limb in individuals with type 2 diabetes mellitus may be relatively separate and not influenced by PAD.

In this study, the presence of hypertension was found to be associated with an increased risk of developing ulcers in the lower extremities. Furthermore, it was observed that hypertension exerts its influence on the development of lower limb wounds through its mediation of PAD. According to a study, hypertension could been considered as one of the leading risk factor for the development of PAD on a global scale (24). When blood pressure exceeds normal levels, it exerts excessive force on the blood vessel wall, resulting in damage to the innermost layer of the blood vessel known as the intima (25). This damage also affects the vascular endothelium, leading to the deposition of lipids from the bloodstream onto the blood vessel wall (26). Consequently, the elasticity of the blood vessel is compromised, thereby facilitating the onset and progression of atherosclerosis. Atherosclerosis is a prominent pathological manifestation of PAD. Therefore, hypertension contributes to the development of chronic lower limb ischemia via PAD, ultimately resulting in the formation of ulcers in the lower extremities.

Obesity is a complex and multifaceted chronic condition that is associated with a wide range of related health issues, the exact relationships of which are not yet fully understood. This lack of clarity may be partially attributed to genetic factors (27). It is correlated with metabolic disorders, resulting in a state of nutritional disequilibrium within the body. Individuals who are both obese and have diabetes are significantly more prone to experiencing complications related to diabetic foot conditions compared to those with diabetes but of normal weight (28). Meulendijks reported that obesity can result in concurrent alterations in the hemodynamics of both the macro- and microcirculation in the lower extremities, ultimately leading to the development of a leg ulcer (29). Mao indicated that the consumption of an excessive diet resulting in obesity can contribute to the development of arterial damage and the progression of atherosclerosis. This is primarily attributed to the inflammatory response triggered in vascular endothelial cells (30). The findings of this Mendelian randomization study demonstrated that both higher BMI and PAD as potential exposures can contribute to the development of lower limb ulcers. However, when the mediating effects of PAD were accounted for, it was determined that higher BMI alone cannot independently cause lower limb ulcers. Based on the results of this study, it could be inferred that the pathological mechanism underlying the association between obesity and lower limb ulcers may involve the development of PAD as a consequence of obesity, leading to lower limb artery ischemia and ultimately the formation of leg ulcers. A significant study has provided evidence for the association between obesity and PAD is a substantial cohort conducted in Israel, involving more than 10,000 male participants. And it revealed that individuals who developed new-onset intermittent claudication (IC) had a higher BMI by 0.5 kg/m^2^ in comparison to men who did not experience IC (31).

Elevated levels of uric acid, known as hyperuricemia, serve as the underlying condition for the development of gout; however, it is not solely responsible for its manifestation. Gout only arises when urate crystals accumulate and inflict damage upon bodily tissues. A heightened concentration of uric acid in the bloodstream increases the likelihood of future gout development (32). Furthermore, the formation of gout crystals can lead to the formation of ulcers in the lower extremities. Tophi are described as nodular accumulations of uric acid crystals that form in various soft tissues of the body, potentially causing joint destruction and leading to ulceration and infection. If treatment is delayed or ineffective, this can result in chronic tophaceous gout. The ulceration of tophi, which is a consequence of prolonged inflammation due to the deposition of monosodium urate crystals, can be considered a persistent wound. Individuals with gout are at a higher risk of having other underlying health conditions that can hinder the healing of wounds, such as diabetes, obesity, and peripheral vascular disease (33). Elevated levels of serum uric acid have been associated with endothelial dysfunction, oxidative stress, and inflammation, all of which play a role in the development of PAD. An observational study demonstrated a positive correlation between higher serum uric acid levels and PAD in male hypertensive patients, although this association was not observed in female participants (34). To investigate the potential causal relationship between hyperuricemia and lower limb ulcers mediated by PAD, we employed Mendelian randomization analysis. According to this MR study, elevated levels of uric acid in the blood are associated with PAD, which aligns with findings from observational study. Surprisingly, our study also found that increased levels of serum uric acid did not result in the development of lower limb ulcers. This observation suggests that the occurrence of hyperuricemia alone may not be sufficient for the formation of lower limb ulcers, as other contributing factors may be necessary. This may necessitate additional histological diagnosis utilizing GWAS and further MR studies to evaluate the correlation between hyperuricemia and lower limb ulcers.

PAD elevates the susceptibility to diabetes, and conversely, diabetes heightens the susceptibility to PAD. Additionally, specific symptoms associated with each condition contribute to an increased risk of developing the other (35). If type 2 diabetes is not effectively managed, it can advance to diabetic foot, a complication characterized by microangiopathy of the skin. This condition can manifest as local cyanosis and ischemic skin ulcers, particularly in the anterior tibia and foot of the lower extremities. The ulcers are typically superficial and cause discomfort, although the dorsal foot artery maintains normal pulsation. However, as the disease progresses, it may also be accompanied by peripheral artery disease. In this study, it was observed that type 2 diabetes may serve as an exposure factor to the development of lower limb ulcers. Additionally, type 2 diabetes could be considered as a potential risk factor for PAD. However, even after controlling for the effect of PAD, type 2 diabetes remained associated with the occurrence of lower limb ulcers. These findings suggest that the etiology of lower limb ulcers in individuals with type 2 diabetes is relatively autonomous and not solely influenced by the presence of PAD.

Our research has several limitations. First of all, the Mendelian randomization approach we employed considers the cumulative lifelong impact of genetic variants and should not be extrapolate to infer the effect of a clinical intervention. In addition, the potential for reverse causation, where PAD may lead to increased BMI or hypertension, resulting in lower limb ulcers, cannot be entirely discounted. We did not investigate the bidirectional relationships as PAD was treated as a binary phenotype, and using such binary exposure may not fully capture the genuine causal relationship in MR analysis. What is more, the genetic association estimates for metabolic syndrome-related diseases and lower limb ulcers were derived from self-reported data, which may be susceptible to recall bias, potentially influencing the MR estimates. Last but not least, the genetic association estimates obtained from the Finnish and EBI databases, which were utilized in this study, represent a specific group and may not be generalizable to broader populations, particularly non-European populations.

In conclusion, this MR study elaborated on the causal risk impact of metabolic syndrome related diseases on the risk of lower limb ulcers. This study adds causal evidence to the etiology of lower limb ulcers and informs prevention and intervention targets to curb the lower limb ulcers epidemic and its related disease burden.

## Data availability statement

Summary statistics on hypertension, BMI, Serum uric acid levels and type 2 diabetes were obtained from the European Bioinformatics Institute (EBI) database, and data on PAD, and ulcers of lower limb were obtained from the Finnish database. The original contributions presented in the study are included in the article/**Supplementary Material**. Further inquiries can be directed to the corresponding authors.

## Ethics statement

Ethical approval was not required for the study involving humans in accordance with the local legislation and institutional requirements. Written informed consent to participate in this study was not required from the participants or the participants’ legal guardians/next of kin in accordance with the national legislation and the institutional requirements. Written informed consent was obtained from the individual(s) for the publication of any potentially identifiable images or data included in this article.

## Author contributions

HW: Conceptualization, Data curation, Formal Analysis, Investigation, Methodology, Project administration, Resources, Software, Validation, Visualization, Writing – original draft, Writing – review & editing. ZeZ: Conceptualization, Data curation, Formal Analysis, Investigation, Methodology, Resources, Software, Validation, Visualization, Writing – original draft. LXZ: Investigation, Software, Writing – original draft. JZh: Methodology, Supervision, Writing – original draft. ZJ: Investigation, Visualization, Writing – original draft. LZ: Investigation, Supervision, Validation, Writing – review & editing. XH: Investigation, Validation, Visualization, Writing – original draft. XS: Data curation, Investigation, Resources, Software, Writing – review & editing. JZo: Conceptualization, Data curation, Formal Analysis, Investigation, Methodology, Project administration, Resources, Software, Supervision, Validation, Writing – review & editing. ZnZ: Conceptualization, Investigation, Methodology, Software, Supervision, Validation, Visualization, Writing – review & editing. SW: Conceptualization, Data curation, Formal Analysis, Investigation, Methodology, Project administration, Resources, Software, Supervision, Validation, Visualization, Writing – original draft, Writing – review & editing.
