# Supplementary figures and images for: Peripheral artery disease mediating the effect of metabolic syndrome related diseases on lower limb ulcers: Mendelian randomization analysis
Source: Front Endocrinol (Lausanne). 2024 Feb 15;15:1345605. doi: 10.3389/fendo.2024.1345605 (PMC10905962; doi:10.3389/fendo.2024.1345605)

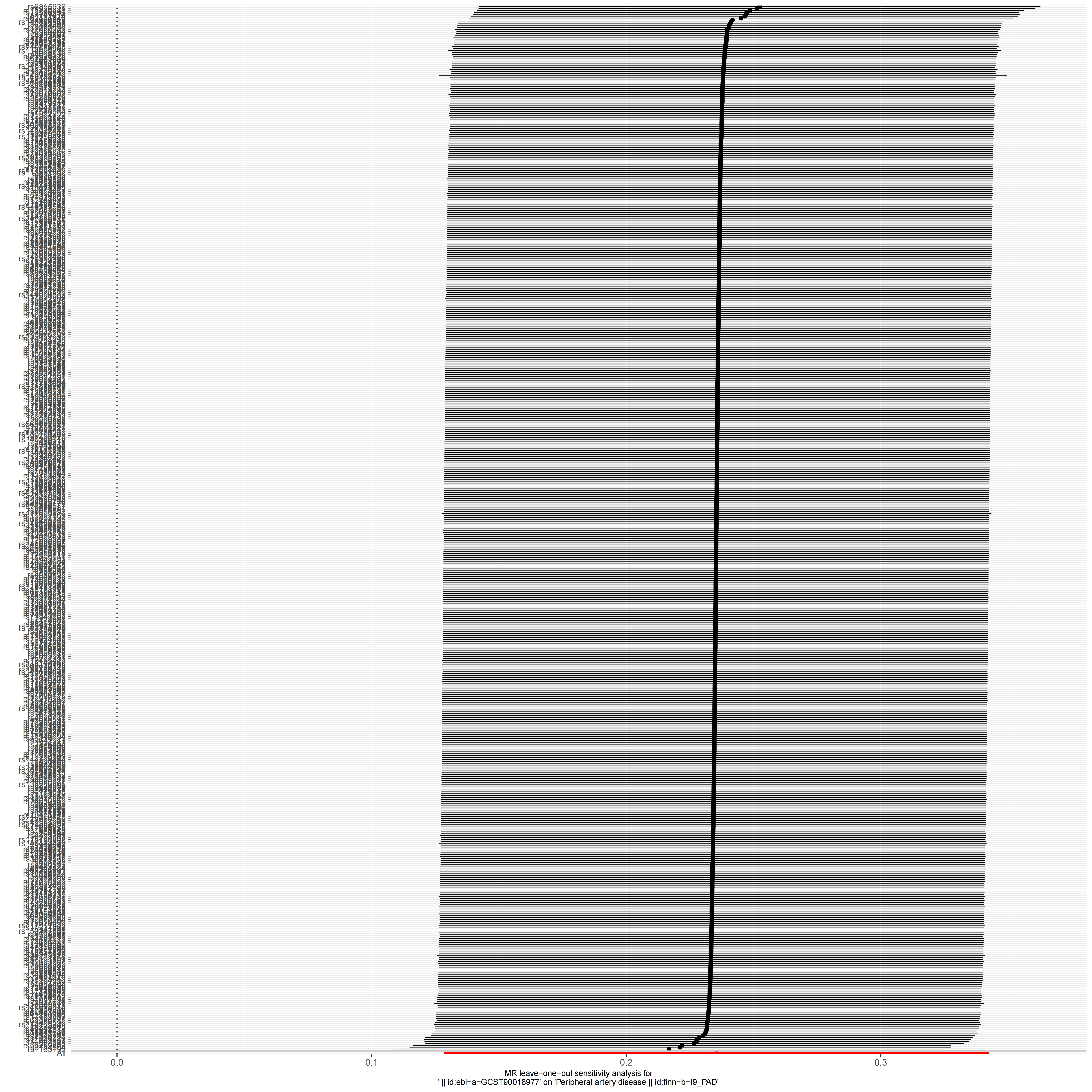

Supplement: Supplementary file 1 [file DataSheet_1.zip › Supplementary/Supplementary document 1/Leave-one-out∩╝ÜSerum uric acid levels on PAD.png]
